# Biometric indicators of eyes with occult lens subluxation inducing secondary acute angle closure

**DOI:** 10.1186/s12886-020-01355-7

**Published:** 2020-03-05

**Authors:** Xiaoli Xing, Liangyu Huang, Fang Tian, Yan Zhang, Yingjuan Lv, Wei Liu, Aihua Liu

**Affiliations:** Tianjin Medical University Eye Hospital, Tianjin Medical University Optometry College, Tianjin Medical University Eye Institute, 251 Fukang Road, Nankai District, Tianjin, 300384 China

**Keywords:** Lens subluxation, Acute angle-closure, Biometry, Anterior chamber depth, Lens thickness, Axial length

## Abstract

**Background:**

To compare the anterior biometrics in eyes with secondary acute angle closure induced by occult lens subluxation (ASAC-LS), misdiagnosed as acute primary angle closure (APAC) at the first visit with APAC, chronic primary angle closure glaucoma (CPACG), and cataract.

**Methods:**

This retrospective case study included 17 eyes with angel closure due to occult LS, who were misdiagnosed as APAC on their first visit, 56 APAC eyes, 54 CPACG eyes, and 56 cataract eyes. Axial length (AL), central corneal thickness (CCT), anterior chamber depth (ACD), aqueous depth (AD) and lens thickness (LT) were recorded. Lens position (LP), relative lens position (RLP), corrected lens position (CLP) were calculated. Quantitative data were subject to one-way analysis of variance and correlation analysis. Categorical data were analyzed using the chi-squared test. Receiver operating characteristic (ROC) curves were plotted to obtain a suitable cutoff value of ocular biometrics.

**Results:**

The ASAC-LS patients had a longer ocular axial length than APAC and CPACG patients. Central corneal thickness of the ASAC-LS patients was not significantly different from APAC patients, but was significantly different from CPACG and cataract patients. The APAC patients had the smallest ACD, while the ASAC-LS patients had the smallest AD. The ASAC-LS patients had the largest lens thickness. According to ROC curve analysis, RLP, ACD, AD, CLP, LP had high power of discrimination.

**Conclusions:**

This study revealed that LS secondary PAC patients had a shallower AD, thicker CCT comparing to those of APAC, CPACG and cataract patients. For patients with acute angle-closure glaucoma, it is necessary to exclude lens zonula relaxation.

**Trial registration:**

NCT03752710, retrospectively registered.

## Background

Lens subluxation (LS) refers to a common malpositionof lens, whose pathological mechanism involves partial zonular dehiscence of lens, causing partialdeviation of the lens from its original position.LS is a common cause of acute secondary angle closure, which is an ophthalmic emergency that can often lead to irreversible optic nerve damage and requires timely treatment to counteract elevated intraocular pressure (IOP) [1]. Acute secondary angle closureinduced by LS (ASAC-LS) is very similar to acute primary angle closure (APAC). They both manifest as acute attacks including severe pain in the eye, headache, ocular hypertension, and shallow anterior chamber. Nonetheless, the clinical manifestations of occult LS are atypical, with insignificant signs ofiridodonesis and phacodonesis. Whenacute secondary angle closure is misdiagnosed as APAC, miotic agents might be administered, andtrabeculectomy may even beperformed. However, if correct diagnosis can be made before the surgery, appropriate treatmentswould be applied according to the patients’ condition, thereby increasing the success rate of the surgery andthe recovery of visual function [2].

The biometric characteristics of patients with APAC includesmall corneal diameter, short ocular axial length, shallow central and peripheral anterior chamber [3, 4], lens thickening [5], and anterior displacement of the lens. To this end, we analyzed anterior segment biometric characteristics in a group of patients with ASAC-LS,and compared these biometric characteristics withthe patients with APAC and chronic primary angleclosure glaucoma (CPACG). It is hoped that this study can be helpful for the diagnosis and differential diagnosis of these ocular diseases.

## Methods

This retrospective case study included 17 eyes of 17 patients with ASAC-LS (7 left eyes and 10 right eyes), who were misdiagnosed as APAC on their first visit. All of the patients were admitted from Jan 10, 2016 to Dec 28, 2017 in Tianjin Medical University Hospital. In addition, sample size of the control groups was calculated and comparing with the ASAC-LS group, we chose 3:1 ratio. Clinical features and demographic of the patients were retrieved from the patient’s medical records from Jan 10, 2016 to Mar, 2016. 56 eyes of 56 patients diagnosed with APAC, 54 eyes of 54 patients diagnosedwith CPACG, and 56 eyes of 56 patients diagnosed with cataract in patientswere also consecutively included (All the patients had vision in both eyes). All data were collected after the cornea were clear before surgeries. This study was conducted in accordance with the Helsinki Declaration and was approved by the Ethics Committee of Tianjin Medical University Eye Hospital.

ASAC-LSwas diagnosed according to the following criteria, including sudden pain in the eye,decreased vision with or without nausea and vomiting. Slit lamp microscopy reveals phacodonesis,lens inclination or vitreous herniation into the anterior chamber, central and peripheral shallow anterior chamber,and asymmetric iris bulge. The diagnosises ofLS were confirmed during the surgery. The range of lens zonula dehiscence was recorded during subsequent surgery, with the average of 5 ± 2.24 o’clock lens zonula dehiscence.

APAC was diagnosed with the following criteria [6–8], including substantially elevated IOPand closed angle, acute eye pain, blurred vision,or nausea and vomiting. More importantly, ischemic injury caused by acute ocular hypertension, ciliary or mixed congestion, corneal edema, and glaucoma flecks should be detected.

The diagnostic criteria of CPACG included narrow angle with anterior synechiae of varying widths, IOP > 22 mmHg, and glaucomatous optic disc damage and visual field defect [9–11]. The angle closure should be more than two quadrants, yet there was no ischemic injury in the anterior segment caused by acute ocular hypertension.

Exclusion criteria were history of laser peripheral iridotomy or peripheral iridectomy, glaucoma filtration surgery,angle closure caused by ocular trauma, uveitis, myopia (more than -3D), neovascularization or intumescentswelling or hypermature lens. The patients with acute angle closure in both eyes were excluded. The subjects whose Lenstar LS 900**(**Haag-Streit USA, INC., USA) results were not available because of severe lens opacity or corneal edema were also excluded.

All patients underwent detailed ocular examinations, including visual acuity, slit lamp microscopy, Goldmann IOP measurements, fundus examinations, Goldmann gonioscopy, Lenstar LS900 biometric measurements. Ocular biometric parameters including ocular axial length (AL), central corneal thickness (CCT), aqueous depth (AD, depth from the endothelium of the cornea to the anterior surface of the lens), anterior chamber depth (ACD, depth from the epithelium of the cornea to the anterior surface of the lens, equal to CCT + AD), and lens thickness (LT) were recorded. Ultrasound biomicroscopy (UBM) were performedby an experienced ophthalmologist to evaluate the lens position as described in the literature [2]. The following formulas were used to assess the biometric chataracters of the patients. Lens position (LP) = ACD + 1/2LT. Corrected anterior chamber depth (CLP) = AD+ 1/2LT. Relative lens position (RLP) = [ACD + 1/2LT]/AL × 10 [12]. To investigate the relevant factors of LS induced acute IOP elevation, we analyzed the correlation of the AD and the range of lens zonula dehiscence statistically.

### Statistical analysis

Statistical analysis was performed using SPSS version 18.0 (IBM Corporation, Armonk, NY, USA). Quantitative data wereexpressed as mean ± standard deviation. Quantitative data were subject to one-way analysis of variance and correlation analysis. Categorical data were analyzed using the Chi-squared test, and *P* < 0.05 was considered to be statistically significant.

Receiver operating characteristic (ROC) curves were plotted to obtain a suitable cutoff value of ocular biometry to separate ASAC-LS patients from APAC by Stata 13.0 (Stata Corp LLC, College Station, TX, USA). *P*-value < 0.05 was considered statistically significant.

## Results

Biometric parameters and multiple comparisons among the four groups were recorded in Tables 1 and 2, respectively. As shown in Table 1, constituent ratio of gender were different statistically and there were no statistically significant difference in terms of age among 4 groups. Except for AL and CCT, the differences of the other measured parameters between cataract group and each of other groups were statistically significant (*P* < 0.05, respectively). As shown in Table 2,AD was a sensitive indicator because there were significant differences between each of the two groups. Compared with APAC and ASAC-LS, AL, ACD, AD, LP, CLP, RLP were different statistically.

**Table 1:** Biometry parameters in groups

	Parameters				Pvalue
	ASAC-LS	APAC	CPACG	Cataract	
Gender					0.025*
Male	11	12	16	20	
Female	7	44	38	36	
Age (y)	64.47 ± 7.82	66.05 ± 8.41	67.44 ± 7.97	67.61 ± 11.14	0.540
AL (mm)	23.23 ± 0.68	22.42 ± 0.77	22.56 ± 0.92	23.47 ± 1.30	0.000*
CCT (μm)	569.00 ± 91.66	552.98 ± 40.29	527.57 ± 39.24	536.46 ± 37.29	0.002*
AD (mm)	1.25 ± 0.35	1.64 ± 0.26	1.77 ± 0.22	2.59 ± 0.39	0.000*
ACD (mm)	2.49 ± 0.56	2.21 ± 0.26	2.33 ± 0.32	3.13 ± 0.39	0.000*
LT (mm)	5.13 ± 0.41	4.97 ± 0.30	4.92 ± 0.30	4.48 ± 0.41	0.000*
LP	4.39 ± 0.32	4.69 ± 0.21	4.79 ± 0.33	5.37 ± 0.27	0.0008
RLP	1.89 ± 0.14	2.09 ± 0.09	2.12 ± 0.16	2.29 ± 0.12	0.000*
CLP	3.82 ± 0.33	4.13 ± 0.21	4.23 ± 0.19	4.83 ± 0.28	0.000

*:*P* < 0.05

**Table 2:** Comparison of biometry parameters in different groups

Mean difference (*P*-value)						
	APAC vs ASAC-LS	CATA vs ASAC-LS	CPACG vs ASAC-LS	CATA vs APAC	APAC vs CPACG	CATA vs CPACG
AL	−0.80827(0.002) **	0.23638(0.902)	−0.66763(0.016) *	1.044643(0.000) **	−0.140642(0.945)	0.904001(0.000) **
CCT	16.01786(0.212)	32.53571(0.012) *	−41.42593(0.001) **	−16.517857(0.147)	25.408069(0.007) **	8.890212(0.780)
ACD	0.388887 (0.001) **	1.31764(0.000) **	0.51779(0.000) **	0.928750(0.000) **	−0.128902(0.127)	0.799848(0.000)**
AD	0.398697 (0.002)**	1.346555 (0.000) **	0.52756(0.000) **	0.947857(0.000) **	−0.128862(0.035) *	0.818995(0.000) **
LT	−0.17181(0.526)	−0.65895(0.000)**	− 0.22342(0.256)	−0.487143(0.000) **	0.051614(0.936)	−0.435529(0.000)**
LP	0.305305 (0.009) **	0.98816(0.000) **	0.40608(0.001) **	0.682857(0.000) **	−0.100774(0.323)	0.582083(0.000) **
CLP	0.31512(0.009)**	1.01708(0.000)**	0.41585(0.001)**	0.701964(0.000) **	−0.100734(0.058)	0.601230(0.000) **
RLP	0.20384(0.000)**	0.4047594(0.000)**	0.23708(0.000)**	0.200923(0.000) **	−0.033242(0.678)	0.167681(0.000) **

The results of ROC curve analysis for each biometric parameters were presented in.

**Fig. 1:**
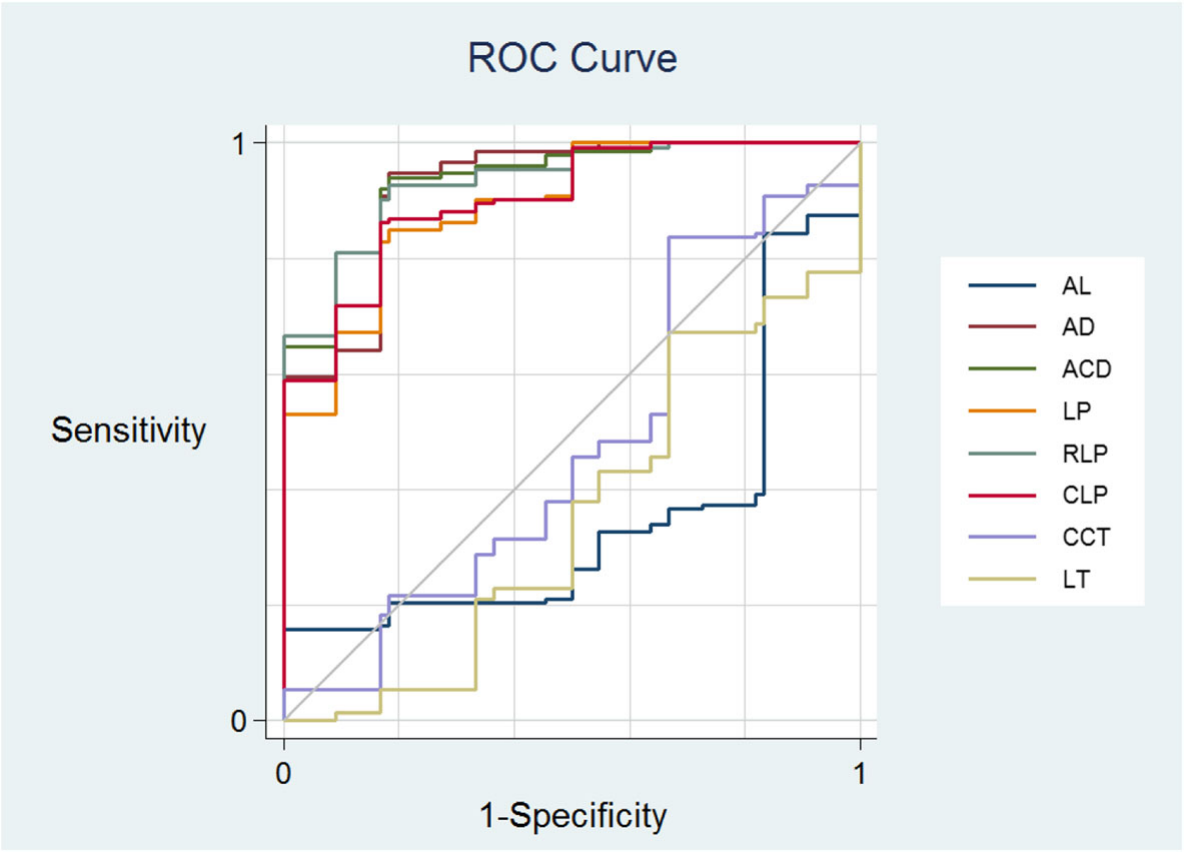
ROC curves plotting sensitivity against one-specificity adjusted for gender. In our study, RLP is the best value to distiguish APAC from ASAC-LS

Figure 1 and Table 3. The results of ROC: RLP (AUROC: 0.934), ACD (AUROC: 0.929), AD (AROC: 0.925), LP (AUROC: 0.892), CLP (AUROC: 0.903).

**Table 3:** Area under the receiver operating characteristic curve (AUROC), sensitivity, specificity, and cutoff value in ASAC-LS and APAC subjects

VALUE	AUC	95%CI	Cut off value
AD	0.928	0.845–1.001	2.16
ACD	0.933	0.867–0.999	2.67
LP	0.896	0.813–0.979	4.87
RLP	0.936	0.879–0.993	2.27
CLP	0.906	0.831–0.981	4.37
AL	0.370	0.214–0.527	24.19
CCT	0.470	0.274–0.665	603
LT	0.340	0.156–0.524	5.23

(LP = ACD + 1/2LT, RLP = [ACD + 1/2LT] /AL × 10, CLP = AD+ 1/2LT. *P* < 0.05 was considered statistically significant)

## Discussion

The main mechanism of PACG is considered as pupillary block. Increased resistance of aqueous humor flow between the iris and anterior lens surface leads to angle closure. A short axial length (AL), thick lens, anteriorly placed lens, are the main risk factors [13].

Traumatic or spontaneous lens dislocationcan cause acute angle closure. The features of zonular instability include iridodonesis, decentration of the nucleus, phacodonesis, the lens equator exposure, and vitreous prolapse in the AC. In clinic, due to the risk of iatrogenic angle-narrowing and elevated intraocular pressure, angle-closed eyes usually do not undergo pharmacologic pupil dilation [14]. Due to relaxation or lens zonula dehiscence, the anterior capsule of the lens can attach or adhere to the posterior surface of the iris [1]. The lens and/or vitreous herniation can cause pupil block, leading to an increase in posterior chamber; consequently, the iris is pushed forward and anterior angle will be closed, resulting in increased IOP. Its clinical manifestations are very similar to those of APAC and, thus, is prone to misdiagnosis. The literature also suggested that the major form of the secondary glaucoma associated with lens subluxation was the open-angle type [15].

We analyzed the clinical features of a group of patients with acute secondary angle closure due to lens dislocation, monocular onset, and acute anterior chamber shallowing. Compared with the APAC, CPACG, and cataract groups, the anterior chamber of patients with acute angle closure due to lens dislocation was significantly shallower, even less than 0.66 mm. The result showed that AD was a sensitive indicator, because it wasstatisticlly significant inall groups’ individually comparison. Therefore, whenever shallow anterior chamber is observed during clinical diagnosis of patients with APAC, it is necessary to devote attention to acute secondary angle closure caused by lens factors. Considering the constituent ratio of gender were different in four groups (*p* < 0.05), we used ROC adjusted of gender to distinguish which factor was the most sensitive one. The result showed that RLP was the most sensitive one to distingush ASAC-LS patinets from the other three groups. Relative lens position (RLP) = [ACD + 1/2LT] /AL × 10.This formula has three parameters, ACD, LT, AL which we should focused on in clinic. According to our results, LT and AL were not sensitive vaue to distinguish these 4 diseases. It has been reported that the anterior chamber depth is significantly different between the involved eye and the contralateral eye in patients with acute angle closure due to LS [16]. The calculated parameters - RLP,LP, CLP showed significant difference in multiple comparison results and were sensitive indicators of four groups.

The RLP (AUROC: 0.934), ACD (AUROC: 0.929), AD (AROC: 0.925), LP (AUROC: 0.892), CLP (AUROC: 0.903), had high power of discrimination. LT in our study was not a sensitive value to distiguish APAC from ASAC-LS. While in primary angle closure patients, LT was a powerful value [12].

In this study, data from the contralateral eyes were incomplete; therefore, anterior chamber depth was not compared between the two eyes.

Patients with angle-closure glaucoma usually exhibit a shorterocular axial length. However, the ocular axial length in the group of patients with acute angle closure caused by ASAC-LS was not significantly different from that in the cataract group, but was longer than that in the APAC and CPACG groups. It has been reported that LS patients have the longest ocular axial lengthamong the population with acute angle closure. Other causes of acute angle closure include iris bombe, pupil block, and plateau iris [17, 18].

Among the four groups of patients, lens thickness in the ASAC-LS group was the greatest, and was significantly different from that in the cataract and the CPACG groups; therefore, lens thickness was not sufficientto diagnose the four diseases. As a result, lens position (LP) (defined as the sum of anterior chamber depth and 1/2lens thickness) was introduced in this study. Calculations indicated thatthere was significant difference between any two groups. Some studies [19] in the literature used lens vault (defined as the perpendicular distance between the anterior lens pole and the horizontal line joining the two scleral spurs) measured using UBM as an indicator of lens morphologyand found that lens vault increases in patients with unstable suspensory ligaments of the lens.

When the lens is subluxated, the lens zonule dehisencehas a large effect on the position of the lens. In this group, the dehisence was recorded during surgery and was found to correlate withAD. Therefore, for occult LS, which does not have clear clinical manifestations and does not have a very high UBM diagnosis rate in our data.

The diagnostic accuracy was 98.0% with 25 MHz UBM and slightly subluxated lens eyes could be detected [20].

AD can be used as one of the indirect determinant indicators.

In summary, we retrospectively analyzed biometric characteristics of the anterior segment of patients with acute angle closure secondary to occult LS. Several points should be addressed during diagnosis and treatment. For youngerpatients with acute angle-closure glaucoma, it is necessary to exclude lens zonula relaxation caused by abnormal lens development; otherwise, the patients would bemisdiagnosed with APAC rather than acute secondary angle-closure glaucoma due to lens dislocation and undergo peripheral iridotomy or glaucoma filtering surgery, which not only increases the risk for complications, such as intraoperative vitreous herniation, postoperative shallow anterior chamber and even malignant glaucoma, but also reduces the success rate of the operation. When applicable, UBM should be used to observe whether the suspensory ligament of the lens is severed or simply relaxed. The anterior chamber depth should be measured: a short depth (< 1.25 mm) is highly indicative of abnormality in the lens zonuladehiscence or relaxation, andthe depth should be compared with that of the contralateral eye. Lens thickness should be measuredand, if it is > 5.13 mm, abnormal suspensory ligament of the lens should be suspected. Meanwhile, LP and CLP can be calculated for differential diagnosis.

Limitations of the present study include the absence of a biometric comparison of the lateral eyes in each group of patients. LS900 can only be used to measure the patients with no serious opacity of cataracts. The gender difference was a factor which may introduce biases. Due to the small number of samples in our study, we will discuss it in the future study.

## Conclusions

For patients with acute angle-closure glaucoma, it is necessary to exclude lens zonula relaxation. A short depth (< 1.25 mm) and a thick lens thickness (> 5.13 mm) would be considered strong predictors for acute angle closure crisis. LP and CLP can be helpful for differential diagnosisbetween angle closed glaucoma and cataract.

## Data Availability

The datasets used and/or analysed during the current study are available from the corresponding author on reasonable request.

## Abbreviations

ACD: Anterior chamber depth
AD: Aqueous depth
AL: Axial length
APAC: Acute primary angle closure
ASAC-LS: Acute secondary angle closure induced by len subluxation
CATA: Cataract
CCT: Central corneal thickness
CLP: Corrected anterior chamber depth
CPACG: Chronic primary angle closed glaucoma
IOP: Intraocular pressure (IOP)
LP: Lens position
LS: Lens subluxation
LT: Lens thickness
RLP: Relative lens position
UBM: Ultrasound biomicroscope
